# N-Terminal Pro-Brain Natriuretic Peptide Correlates with Ghrelin and Acyl-Ghrelin in Pre-Dialysis Chronic Kidney Disease

**DOI:** 10.3390/ijms25115696

**Published:** 2024-05-23

**Authors:** Crina Claudia Rusu, Florin Anton, Ana Valea, Cosmina Ioana Bondor

**Affiliations:** 1 Department of Nephrology, University of Medicine and Pharmacy “Iuliu Hatieganu”, 400012 Cluj-Napoca, Romania; 2 “Mihai Manasia” Nephrology and Dialysis Clinic, County Emergency Clinical Hospital Cluj, 400347 Cluj-Napoca, Romania; 3 Department of Cardiology, University of Medicine and Pharmacy “Iuliu Hatieganu”, 400012 Cluj-Napoca, Romania; 4 Cardiology Clinic, County Emergency Clinical Hospital Cluj, 400347 Cluj-Napoca, Romania; 5 Department of Endocrinology, University of Medicine and Pharmacy “Iuliu Hatieganu”, 400012 Cluj-Napoca, Romania; 6 Endocrinology Clinic, County Emergency Clinical Hospital Cluj, 400347 Cluj-Napoca, Romania; 7 Department of Medical Informatics and Biostatistics, University of Medicine and Pharmacy “Iuliu Hatieganu”, 400349 Cluj-Napoca, Romania

**Keywords:** heart failure, biomarkers, pro-brain natriuretic peptide, ghrelin, acyl-ghrelin, chronic kidney diseases

## Abstract

Pro-B amino-terminal natriuretic peptide (NT-proBNP) is a diagnostic marker for heart failure (HF), a severe complication of chronic kidney disease (CKD). However, its significance in CKD is not clear, as other factors, such as renal function, may also have an impact. Recent studies have shown that ghrelin treatment is effective in HF in the general population, but the impact of ghrelin on cardiac function in CKD patients is still unknown. Our study aimed to investigate the factors associated with NT-proBNP in pre-dialysis CKD patients and to evaluate the correlation between NT-proBNP and ghrelin and acyl-ghrelin, molecules determined using ELISA methods. In a cross-sectional observational study, we included 80 patients with pre-dialysis CKD, with a mean age of 68 years and 50% men. The median values for NT-proBNP were 351.8 pg/mL, for acyl ghrelin 16.39 pg/mL, and for ghrelin 543.32 pg/mL. NT-proBNP was correlated with ghrelin (*p* = 0.034, r = 0.24), acyl-ghrelin (*p* = 0.033, r = −0.24), estimated glomerular filtration rate (*p* = 0.027, r = −0.25), serum urea (*p* = 0.006, r = 0.31), and ferritin (*p* = 0.041, r = 0.28). In multivariate analysis, ghrelin (*p* = 0.040) and blood urea (*p* = 0.040) remained significant predictors for NT-proBNP levels. NT-proBNP was a significant predictor for acyl-ghrelin (*p* = 0.036). In conclusion, in pre-dialysis CKD patients, a high value of NT-proBNP was associated with a high value of total ghrelin and a low value of acyl-ghrelin.

## 1. Introduction

Chronic heart failure (HF) affects 25% of pre-dialysis chronic kidney disease (CKD) patients, and its prevalence increases as the disease progresses, reaching up to 65–70% in end-stage renal disease [1], and it has a major impact on patient survival [2]. It is essential to assess the diagnostic and prognostic biomarkers for HF in CKD patients. Additionally, it is crucial to propose novel treatment options for this disease. Natriuretic peptides are molecules linked to myocardial dysfunction and are known as biomarkers. Among them, B-type natriuretic peptide (BNP) and pro-B amino-terminal natriuretic peptide (NT-proBNP) are commonly tested [3] and are important indicators for diagnosis, prognosis, and treatment monitoring in chronic HF. NT-proBNP is superior to BNP as a prognostic value [4,5,6,7]. After being secreted from the coronary sinus, BNP and NT-proBNP are either enzymatically degraded by neutral endopeptidase and dipeptidyl peptidase-4 or excreted by the kidneys [8]. NT-proBNP is mainly cleared through glomerular filtration by the kidney, which explains the strong correlation between renal function and NT-proBNP levels [9]. In patients with CKD, besides heart and renal failure, other factors, such as hyperhydration, left ventricular hypertrophy, anemia [3,10], age, inflammation, and malnutrition, can increase the levels of NT-proBNP [11,12,13,14,15]. In these patients, the ratio between the cardiac secretion of NT-proBNP linked to different stimuli and the retention of NT-proBNP due to renal dysfunction is unknown [16]. Thus, the level of NT-proBNP has a lower specificity and sensitivity for the diagnosis of acute HF in CKD 3–5 stages [17]. Fu et al. suggest that NT-proBNP depends on cardiac function in CKD patients with an estimated glomerular filtration rate (eGFR) of 45–60 mL/min/1.73 m^2^, while for an eGFR < 45 mL/min/1.73 m^2^, it depends on renal function [18]. There are no established NT-proBNP values for diagnosing HF in patients with CKD [19]. However, NT-proBNP is an independent predictor of mortality in CKD patients, and it can also be useful for cardiovascular risk stratification in these patients [20], being significantly associated with the risk of incident HF [21].

The high prevalence and severity of HF in pre-dialysis CKD patients may be explained by hormonal imbalances (such as ghrelin, prolactin, etc.) and the specific inflammatory syndrome associated with CKD. In this case, certain molecules that act as markers of HF in CKD may be linked to hormone levels, such as ghrelin or prolactin levels. Identifying such associations could have therapeutic implications, since ghrelin treatment is effective for HF in the general population, and perhaps we can use this treatment in CKD patients as well.

Ghrelin is an appetite-stimulating hormone secreted in the small intestine and stomach in response to fasting and weight loss, and its activation involves the acylation of amino acid 3 [22,23,24]. There are two major forms of circulating ghrelin: acyl-ghrelin, which is orexigenic, and des-acyl ghrelin, which has possible anorexigenic effects [25,26,27,28,29]. Ghrelin is primarily metabolized and excreted by the kidneys. Several studies have found that patients with CKD have higher levels of circulating ghrelin than the general population. This is because the kidneys are less able to break down ghrelin in people with CKD. Other factors, such as nutritional status, inflammation, age, and sex, can also influence ghrelin levels in CKD patients [30,31,32]. Ghrelin has several functions, including carbohydrate and energy metabolism and gastrointestinal, cardiovascular, pulmonary, and immune functions. It can also stimulate osteoblast proliferation and bone formation [33]. Reduced levels of total ghrelin and acyl-ghrelin in end-stage kidney disease patients are associated with higher rates of mortality and cardiovascular morbidity, particularly when combined with inflammatory and nutritional markers [34,35]. Studies on chronic hemodialysis patients indicate that higher levels of acyl-ghrelin are associated with higher body mass index (BMI) and better survival, regardless of appetite, nutritional status, and inflammation [36]. High levels of NT-proBNP were correlated with elevated levels of ghrelin in obese dialysis patients [37] and with low levels of acyl-ghrelin in male hemodialysis patients [38]. To the best of our knowledge, no studies have examined the relationship between ghrelin/acyl-ghrelin and NT-proBNP in pre-dialysis CKD stages. 

In CKD patients, the level of prolactin is higher than in the general population. This is due to reduced renal elimination and increased production caused by decreased sensitivity to dopaminergic inhibition [39]. Hyperprolactinemia has been associated with general and cardiovascular morbidity and mortality in CKD patients [40,41].

Among the markers of inflammation associated with cardiovascular diseases in pre-dialysis CKD patients, the role of cytokines such as interleukin 1 beta (IL-1 beta) [42] is known, but their relationship with NT-proBNP or hormonal status has not been studied.

This study aimed to evaluate the factors that can influence the level of NT-proBNP in pre-dialysis CKD patients, especially the relationship between NT-proBNP and endogenous ghrelin, and acyl-ghrelin levels.

## 2. Results

### 2.1. Patients’ Characteristics

We recorded clinical and laboratory data for patients with CKD, and we observed an equal number of women: men, 1/3 patients with diabetes, a mean age over 65 years, and almost all patients with hypertension. Parameters that may influence cardiovascular disease in CKD patients were also recorded. These included blood pressure values; nutritional markers such as adipose tissue mass, lean tissue mass, body mass index (BMI), serum albumin, lipid fractions, and fasting glucose; markers of mineral and bone metabolism, including serum calcium, phosphorus, alkaline phosphatase, and intact parathormone; markers of the inflammatory syndrome; the level of hemoglobin; and renal function expressed by the estimated glomerular filtration rate. Hormonal markers, including NT-proBNP, were also studied.

Demographical, clinical, and laboratory patient characteristics are presented in Table 1. 

All patients were Caucasian. No patient included was under angiotensin receptor/neprilysin inhibitor (ARNI) medication.

### 2.2. Determinants of NT-proBNP 

**Figure 1:**
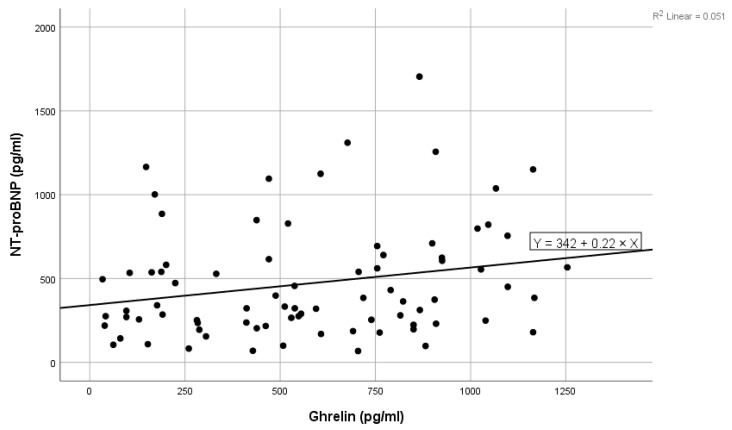
Positive linear correlation between NT-proBNP and ghrelin in the total group.

**Figure 2:**
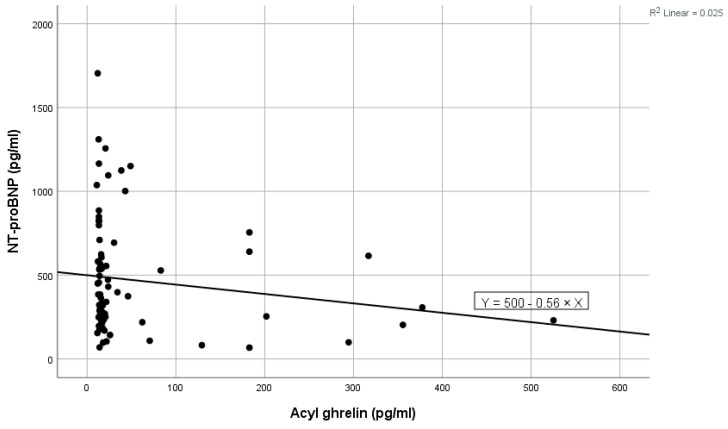
Negative linear correlation between NT-proBNP and acyl-ghrelin in the total group.

High values of NT-proBNP were statistically significantly associated with high values of ghrelin (shown in Figure 1), high levels of blood urea, high levels of ferritin, and high values of LDL cholesterol, and near statistically significantly correlated with high prolactin values. Low levels of NT-proBNP were statistically significantly associated with high levels of acyl-ghrelin (shown in Figure 2) and with high levels of eGFR (Table 2). In the multivariate analysis, it was noted that only ghrelin levels and blood urea remained significantly associated with NT-proBNP (Table 2).

Using receiver operator curve analysis for NT-proBNP, the concentration of 287.35 pg/mL (the area under the curve = 0.65, 95% confidence interval (CI) 0.52–0.78, *p* = 0.029, sensitivity = 0.75, specificity = 0.55) was identified as the optimal cut-off value in relation with eGFR (=30 mL/min/1.73 m^2^). In conclusion, when comparing NT-proBNP for the group with eGFR < 30 mL/min/1.73 m^2^, statistically significantly more subjects had NT-proBNP > 287.35 pg/mL than those in the group with eGFR ≥ 30 mL/min/1.73 m^2^ with NT-proBNP > 287.35 pg/mL [33 (75%) vs. 13 (44.8%), *p* = 0.009]. 

### 2.3. Determinants of Ghrelin 

Regarding ghrelin in the analysis of correlations, it was observed that high values of ghrelin were statistically significantly associated with high values of BMI, adipose tissue mass, triglycerides, fasting glucose, and prolactin. Low levels of ghrelin were statistically significantly associated with lean tissue mass, HDL cholesterol, eGFR, and DBP (Table 3). In the multivariate analysis, it was noted that triglycerides and BMI levels remained significantly associated with ghrelin (Table 3).

### 2.4. Determinants of Acyl-Ghrelin 

For high acyl-ghrelin, we noted correlations with low levels of NT-proBNP and with high levels of IL-1 beta, triglycerides, triglycerides, and serum bicarbonate (Table 4). The multivariate analysis showed that only NT-proBNP remained significantly associated with acyl-ghrelin (Table 4).

## 3. Discussion

Our research found that ghrelin, acyl-ghrelin, prolactin, eGFR, blood urea, ferritin, and LDL cholesterol were correlated with NT-proBNP levels in pre-dialysis CKD patients. After performing multivariate analysis, two molecules, ghrelin and blood urea, remained significant predictors for NT-proBNP. As far as we know, associations between NT-proBNP and ghrelin have not been described previously in this group of patients. The relationship between ghrelin and NT-proBNP has been observed in the general population, but the data are contradictory. In one study, it was noted that high NT-proBNP values were associated with hyperghrelinemia in the elderly and that hyperghrelinemia was associated with severe HF assessed by ultrasound [43]. In another study, researchers found a correlation between high ghrelin levels and low NT-proBNP levels, indicating no association with HF [44]. None of the studies specified which form of ghrelin was being studied: acyl-ghrelin, des-acyl ghrelin, or total ghrelin. This lack of specificity may be a cause of conflicting data. It appears that the acetylated form is required for ghrelin activity [45]. According to a recent review by Hosoda, there are multiple molecules derived from ghrelin, each with a different number of fatty acids. These molecules have been found to inhibit sympathetic activity, stimulate parasympathetic activity, and improve cardiac function in patients with HF by working through growth hormone and insulin growth factor-1 [46]. Not only does ghrelin act through growth hormones, but receptors for ghrelin have also been discovered in the cardiovascular system [47,48]. Experimental studies have shown that ghrelin has a vasodilator effect by acting on calcium-sensitive potassium channels [49] and that ghrelin produces vasodilation on isolated blood vessels precontracted with endothelin [50]. In other research where total ghrelin and acyl-ghrelin were distinguished, the authors noted that total ghrelin can have a maladaptive effect, promoting adipose tissue growth and glucose intolerance [51,52]. In pre-dialysis CKD patients, such an effect may be relevant. In our study, we found that high levels of total ghrelin were associated with high BMI, high adipose tissue mass, high levels of glucose, high levels of triglycerides, high levels of prolactin, reduced levels of HDL cholesterol, and reduced muscle mass, and thus with cardiovascular risk factors. BMI and triglyceride levels were the most significant factors in determining total ghrelin levels in our study. All these correlations of total ghrelin suggest that high levels of total ghrelin are linked to an increased risk of cardiovascular disease. In addition, we observed that total ghrelin levels tend to increase with decreasing kidney function, whereas eGFR does not affect acyl-ghrelin levels. 

The prolactin mentioned above was directly correlated with ghrelin and other atherosclerosis risk factors in our study. It can regulate vessel formation and cardiac remodeling [38], leading to disrupting cardiac angiogenesis, HF, and increasing mortality [53].

As mentioned earlier, the level of NT-proBNP in our study was associated with the blood urea level. In fact, in CKD, the blood urea level is not only a marker of renal function, such as eGFR, but can also be influenced by factors such as appetite, the presence of a hypercatabolic state due to metabolic acidosis, and inflammatory syndrome [54]. Concerning ferritin, it is considered a marker of inflammatory syndrome besides its role in iron metabolism, and probably as an inflammatory marker, it is directly correlated with NT-proBNP in our study, similar to other data in the literature [55].

Regarding acyl-ghrelin, it is known that it has a cardiovascular protective effect due to its antioxidant and anti-inflammatory properties [45]. In our study, we observed that it was linked to inflammatory mediators such as IL-1 beta, but not to classic ones such as high-sensitivity C-reactive protein (hs-CRP). Similarly, previous research conducted on chronic HD patients did not identify any connections between acyl-ghrelin and hs-CRP, TNF alpha, or IL-6 [56]. It was observed in our study that acyl-ghrelin was directly correlated with IL-1 beta; we consider it possible through a compensatory mechanism. It is known that chronic inflammation can affect ghrelin levels in humans and rats. In the rat model of adjuvant-induced arthritis, a compensatory variation in ghrelin level was observed. Similar findings were recorded in patients with rheumatoid arthritis [57]. On the other hand, ghrelin administration can inhibit the expression of pro-inflammatory cytokines, such as IL-1β, IL-6, and TNF-α, which are induced by leptin in human T lymphocytes. It appears that ghrelin and leptin are part of a regulatory network that controls immune cell activation and inflammation. Additionally, ghrelin has potent anti-inflammatory effects and acts as a key signal linking the metabolic axis with the immune system [58].

Low acyl-ghrelin levels were also associated with low levels of serum bicarbonate and therefore with metabolic acidosis, another cardiovascular risk factor in CKD. Acidosis increases endothelin-1 and aldosterone production, furthering CKD progression and cardiovascular pathology [59]. Additionally, our study found that higher levels of acyl-ghrelin are linked to lower levels of NT-proBNP, which suggests that high acyl-ghrelin is linked to good cardiac function. NT-proBNP was found to be the primary determinant of acyl-ghrelin levels in our study, highlighting the importance of the relationship between these two molecules in pre-dialysis CKD patients. Recent studies have shown that administering synthetic acyl-ghrelin can increase cardiac output in individuals with HF and reduce ejection fraction, without significant side effects in the general population [60,61]. Knowing these molecular mechanisms, the relationships that we identified could be the premises of a new treatment for HF in CKD. We have shown that reduced acyl-ghrelin values are associated with increased NT-proBNP values. NT-proBNP is a biomarker of HF with a major predictive role for cardiovascular disease, even in CKD patients. Therefore, the administration of synthetic acyl-ghrelin as a medication can also be discussed to improve cardiac function in these patients. Acyl-ghrelin receptors are widely distributed in cardiac and skeletal muscle and endothelium [47]. In rat HF models, ghrelin increased cardiac output and fractional contractility [62] in a load-independent fashion and without Ca^2+^ mobilization [60]. 

Despite the potential benefits in managing cardiovascular disease that we discussed above in patients with CKD, determining the cut-off value of NT-proBNP for HF diagnosis remains a challenge. Although the current data show that elevated NT-proBNP levels in pre-dialysis and dialysis patients mainly indicate cardiovascular disease and are linked to the risk of future cardiovascular events in CKD [63,64,65], the diagnostic value of elevated NT-proBNP provides moderate or no prediction of heart failure in CKD patients, especially in advanced stages [66,67]. There are no recommendations in the guidelines for cut-off values of NT-proBNP for HF diagnosis in different stages of CKD. Studies have shown that cut-off values for NT-proBNP are greater in CKD [68] and increase as CKD progresses to stage 5, reaching thousands in dialysis patients [69,70,71]. In fact, elevated levels of NT proBNP in CKD may also indicate a high risk of CKD progression in advanced stages [72,73]. In our study, operator curve analysis yielded a 287.35 pg/mL cutoff for NT-proBNP for eGFR less than or greater than 30 mL/min/1.73 m^2^, double the normal laboratory limit of 125 pg/mL. However, we did not look at the advanced stages of CKD.

The results of this study are medically significant, and we can highlight several aspects. Firstly, our study has identified certain molecules that can affect NT-proBNP levels in pre-dialysis CKD patients. Secondly, the study sheds light on the relationship between acyl-ghrelin and NT-proBNP and suggests a new molecular mechanism for HF in CKD. Furthermore, recent studies have shown that acyl-ghrelin administration as medication could improve cardiac function in HF in the general population. In this context, the relationship identified in our study between acyl-ghrelin and NT proBNP could be a basis for acyl-ghrelin treatment in HF in pre-dialysis CKD patients. Additionally, we have remarked on associations that have not been published before in this group of patients. These findings could contribute to better management of HF in CKD patients. 

The study has several limitations. First, it included a relatively small number of patients, so further studies are needed to confirm the correlations and associations between NT-proBNP and ghrelin/acyl-ghrelin levels. Second, there was no control group. Third, the study was observational by design, so the findings need to be confirmed in a prospective interventional study. Fourth, due to the nature of our cross-sectional data, this study was limited in interpreting causality.

## 4. Materials and Methods

### 4.1. Participants

We performed a cross-sectional observational study including patients with pre-dialysis CKD. Of the 82 patients randomized in the Cluj County Emergency Clinical Hospital Department of Nephrology, 80 met the inclusion and exclusion criteria after giving written informed consent. All procedures in the study followed institutional and national research committee ethical standards and the 1964 Declaration of Helsinki and its subsequent amendments. 

The inclusion criteria were age ≥ 18 years, diagnosis of CKD stage 3–5 pre-dialysis, and no kidney transplant for at least six months defined according to the Kidney Disease Improving Global Outcomes guidelines [74], having stable renal function in the last three months (<5 mL/min/1.73 m^2^ change in eGFR), without changes in cardiac medication in the same period. 

The exclusion criteria were the following: acute inflammatory processes, severe neoplasia with a life expectancy of <6 months, chronic or acute diseases that require medication changes, or absence of data. Demographic data, comorbidities (diabetes, hypertension), and medication at enrollment were obtained from medical records. We also registered clinical data: age, weight, height, systolic blood pressure (SBP), and diastolic blood pressure (DBP). Hypertension was diagnosed according to SBP/DBP ≥ 140/90 mmHg as well as the use of relevant medications. We calculated the pulse pressure (PP) using the formula

PP = SBP − DBP (mmHg).(1)

### 4.2. Anthropometric Nutritional Parameters Assessment

Body mass index was calculated using the formula

BMI = weight (kg)/height^2^ (m^2^). (2)

In addition, nutritional status was assessed by bioimpedance using a certified device (manufactured by Fresenius Medical Care, Bad Homburg, Germany), Body Composition Monitor, which recorded lean tissue mass (kg) and adipose tissue mass (kg) [75].

### 4.3. Laboratory Parameters

All laboratory data were collected between 7:00 and 9:00 a.m. after an overnight fast. Current measurements at baseline included serum electrolytes, albumin, urea, creatinine, lipid profile (total cholesterol, triglycerides, HDL cholesterol, LDL cholesterol), blood glucose, inflammatory markers (high-sensitivity C-reactive protein (hs-CRP), ferritin), intact parathyroid hormone (iPTH), hemoglobin, and white blood cells).

The current laboratory data were recorded, and the methods used for determination were the following: serum electrolytes using potentiometry, albumin, urea, creatinine, lipid profile (total cholesterol, triglycerides, HDL cholesterol, LDL cholesterol), blood glucose, ferritin using spectrophotometry, high-sensitivity C-reactive protein (hs-CRP) using latex immunoturbidimetry, intact parathyroid hormone (iPTH) using chemiluminescence, and hemoglobin and white blood cells using impedance spectroscopy, spectrophotometry, and flow cytometry. Interleukin-1 beta, NT-proBNP, ghrelin, and acyl-ghrelin were determined by enzyme-linked immunosorbent assay (ELISA) using commercially available kits similar to those used in other studies (R&D System, Minneapolis, MN, USA). IL-1 beta, NT proBNP, and ghrelin were measured in serum and acyl ghrelin in plasma. For total ghrelin, the catalog number was DY8149-05, and the intra-assay coefficient of variation was 3.6%; for acyl-ghrelin, the catalog number was RA194062400R, and the intra-assay coefficient of variation was 6.9%; for IL1-beta, the catalog number was RAF048R, and the intra-assay coefficient of variation was 5.1%; for prolactin, the catalog number was DKO011 and the intra-assay coefficient of variation was ≤4.0%; for NT proBNP the catalog number was MBS355233. The minimum detectable levels were as follows: less than 6.1 pg/mL for IL-1 beta, 7.8 pg/mL for NT-proBNP, 1.5 ng/mL for prolactin, 100 pg/mL for ghrelin, and 10 pg/mL for acyl-ghrelin. We used the online equation for eGFR based on creatinine, age, sex, and a coefficient for race [76].

### 4.4. Statistical Analysis

Two patients with NT-proBNP > 4000 were excluded; the respective values were considered input errors. The data of 82 patients were analyzed.

The variables measured on quantitative scales were described using the mean or median if they did not follow the normal distribution. The correlation was reported after calculating the Pearson and Spearman correlation coefficients. The Pearson correlation coefficient was reported for linear relationships, and the Spearman correlation coefficient was reported for non-linear ones.

For the variables correlated with NT-proBNP, a cut-off was found to be related to the median NT-proBNP with the help of receiver operator curves. The cut-off was considered to be the value for which the receiver operator curve had the sum of maximum sensitivity and specificity. For each cut-off, the area under the curve, lower and upper limits of the 95% confidence interval (CI) of the area under the curve, sensitivity, and specificity were reported. The same procedure was followed for the cut-off NT-proBNP in relation to the median of the other variables correlated with it.

Multivariate analysis was performed using linear regression. All significantly correlated or almost significantly correlated quantitative variables in the multivariate linear analysis were analyzed.

Two-sided *p*-values were considered. The level of statistical significance was considered to be α = 0.05. Data analysis was performed using SPSS 25.00 version.

## 5. Conclusions

In conclusion, in pre-dialysis CKD patients, total ghrelin and blood urea levels were found to be significant predictors of NT-proBNP level. High NT-proBNP values were associated with low acyl-ghrelin values. Increased ghrelin levels were linked with proatherogenic markers, while decreased acyl-ghrelin values were associated with metabolic acidosis and low IL-1 beta. NT-proBNP was a significant predictor for acyl-ghrelin level in our patients.

## Figures and Tables

**Table 1:** Characteristics of participants (*n* = 80), arithmetic mean ± standard deviation/median (25th; 75th percentile).

Parameter	Group (*n* = 80)
Age (years)	68 (62; 75)
Male, *n* (%)	40 (50.0)
Diabetes mellitus, *n* (%)	32 (40.0)
Hypertension, *n* (%)	65 (88.8)
SBP (mmHg)	144 (126; 162)
DBP (mmHg)	86.89 ± 12.65
PP (mmHg)	59 (45; 73)
eGFR (mL/min/1.73 m^2^)	27 (15; 39.5)
Body mass index (kg/m^2^)	28.65 (26.55; 30.75)
Lean tissue mass (kg)	35 (27.05; 43.6)
Adipose tissue mass (kg)	40.9 (34.25; 47.25)
Total cholesterol (mg/dL)	173 (153.5; 196.5)
LDL cholesterol (mg/dL)	100.16 ± 30.64
HDL cholesterol (mg/dL)	40 (33; 49.5)
Triglycerides (mg/dL)	133 (93; 169)
Fasting glucose (mg/dL)	103 (92; 133.5)
Serum bicarbonate (mmol/L)	19.6 (17.7; 22.3)
Calcium (mg/dL)	9.17 (8.61; 9.61)
Phosphorus (mg/dL)	3.62 (3.1; 4.52)
iPTH (pg/mL)	118.3 (87.5; 207)
Alkaline phosphatase (UI/L)	84 (72; 104)
Hemoglobin (g/dL)	12.27 ± 2.39
Serum albumin (g/L)	3.82 (3.51; 4.19)
Urea (mg/dL)	85 (56.5; 114.5)
Ferritin (ng/mL)	93 (53; 200)
hs-CRP (mg/dl)	0.45 (0.22; 1.19)
White blood cells (no./mm^3^)	7642.83 ± 2322.96
IL-1 beta (pg/mL)	6.92 (6.38; 12.49)
NT-proBNP (pg/mL)	351.8 (232.77; 610.59)
Ghrelin (pg/mL)	543.32 (270.69; 857.88)
Acyl-ghrelin (pg/mL)	16.39 (14.04; 24.85)
Prolactin (ng/mL)	5.64 (3.66; 9.06)
Angiotensin-converting enzyme inhibitor/angiotensin receptor blocker, *n* (%)	36 (45)

SBP: systolic blood pressure; DBP: diastolic blood pressure; PP: pulse pressure; eGFR: estimated glomerular filtration rate; iPTH: intact parathyroid hormone; hs-CRP: high-sensitivity C-reactive protein; IL-1 beta: interleukin-1 beta; NT-proBNP: amino-terminal pro-B-type natriuretic peptide; no.: number.

**Table 2:** The NT-proBNP correlation with other parameters.

Parameter	Univariate Analysis		Multivariate Analysis	
	Coefficient of Correlation	*p*	B Coefficient 95% CI	*p*
Ghrelin (pg/mL)	0.24	0.034	0.30 (0.02; 0.59)	0.040
Acyl-ghrelin (pg/mL)	−0.24	0.033	-	-
Prolactin (ng/mL)	0.21	0.068	-	-
eGFR (mL/min/1.73 m^2^)	−0.25	0.027	-	-
Blood Urea (mg/dL)	0.31	0.006	2.35 (0.11; 4.59)	0.040
Ferritin (ng/mL)	0.28	0.041	-	-
LDL cholesterol (mg/dL)	0.36	0.012	-	-

eGFR: estimated glomerular filtration rate; CI: confidence interval.

**Table 3:** The ghrelin correlation with other parameters.

Parameter	Univariate Analysis		Multivariate Analysis	
	Coefficient of Correlation	*p*	B Coefficient 95% CI	*p*
Body mass index (kg/m^2^)	0.44	<0.001	5.67 (0.18; 11.17)	0.043
Adipose tissue mass (kg)	0.46	<0.001	-	-
Lean tissue mass (kg)	−0.35	0.006	-	-
Triglycerides (mg/dL)	0.32	0.022	0.47 (0.51; 0.88)	0.029
HDL cholesterol (mg/dL)	−0.31	0.039	-	-
Fasting glucose (mg/dL)	0.29	0.021	-	-
eGFR (mL/min/1.73 m^2^)	−0.23	0.043	-	-
Prolactin (ng/mL)	0.32	0.004	-	-
NT-proBNP (pg/mL)	0.24	0.034	-	-
DBP (mmHg)	−0.35	0.004	-	-

eGFR: estimated glomerular filtration rate; NT-proBNP: amino-terminal pro-B-type natriuretic peptide; DBP: diastolic blood pressure; CI: confidence interval.

**Table 4:** The acyl-ghrelin correlation with other parameters.

Parameter	Univariate Analysis		Multivariate Analysis	
	Coefficient of Correlation	*p*	B Coefficient 95% CI	*p*
NT-proBNP (pg/mL)	−0.24	0.033	0.24 (0.02, 0.46)	0.036
IL-1 beta (pg/mL)	0.94	<0.001	-	-
Triglycerides (mg/dL)	0.29	0.044	-	-
Serum bicarbonate (mmol/L)	0.47	0.015	-	-

NT-proBNP: amino-terminal pro-B-type natriuretic peptide; IL-1 beta: interleukin-1 beta; CI: confidence interval.

## Data Availability

The research data that support the findings of this study are not publicly available. Further inquiries can be directed to the corresponding author.
